# Convection-Enhanced Delivery of Carboplatin PLGA Nanoparticles for the Treatment of Glioblastoma

**DOI:** 10.1371/journal.pone.0132266

**Published:** 2015-07-17

**Authors:** Azeem Arshad, Bin Yang, Alison S. Bienemann, Neil U. Barua, Marcella J. Wyatt, Max Woolley, Dave E. Johnson, Karen J. Edler, Steven S. Gill

**Affiliations:** 1 Functional Neurosurgery Research Group, School of Clinical Sciences, Bristol University, Bristol, United Kingdom; 2 Neurological Applications Division, Renishaw Plc, Gloucestershire, United Kingdom; 3 Department of Chemistry, University of Bath, Bath, United Kingdom; National Cheng Kung University, TAIWAN

## Abstract

We currently use Convection-Enhanced Delivery (CED) of the platinum-based drug, carboplatin as a novel treatment strategy for high grade glioblastoma in adults and children. Although initial results show promise, carboplatin is not specifically toxic to tumour cells and has been associated with neurotoxicity at high infused concentrations in pre-clinical studies. Our treatment strategy requires intermittent infusions due to rapid clearance of carboplatin from the brain. In this study, carboplatin was encapsulated in lactic acid-glycolic acid copolymer (PLGA) to develop a novel drug delivery system. Neuronal and tumour cytotoxicity were assessed in primary neuronal and glioblastoma cell cultures. Distribution, tissue clearance and toxicity of carboplatin nanoparticles following CED was assessed in rat and porcine models. Carboplatin nanoparticles conferred greater tumour cytotoxicity, reduced neuronal toxicity and prolonged tissue half-life. In conclusion, this drug delivery system has the potential to improve the prognosis for patients with glioblastomas.

## Introduction

Glioblastomas are the most common, heterogeneous, highly invasive tumours of the brain, and are associated with poor prognosis. Median survival with maximal treatment of surgery, radiation and temozolomide chemotherapy is reported to be 14.6 months [1]. The infiltrative nature of the tumour inevitably leads to reccurance of the original tumour mass [2]. Conventional delivery of chemotherapy suffers from inadequate penetration of the blood brain barrier (BBB), resulting in subtherapeutic drug concentrations at the tumour site [3].

Local administration of chemotherapy following tumour resection facilitates bypass of the BBB, and aims to target the peritumoral sites of tumour invasion and recurrence. The most well known are wafers containing carmustine (Gliadel, Arbor Pharmaceuticals, USA), which are tightly packed into the resection cavity [4]. This type of drug delivery relies upon diffusion and so cannot penetrate deep into the surrounding brain tissue [5]. This strategy is associated with adverse effects such as convulsions, confusion, brain oedema, infection, hemiparesis, aphasia, and visual field defects, and these complications may outweigh any improvement in survival [6].

Convection-enhanced delivery (CED) offers an alternative strategy for direct delivery of drugs into the brain that does not depend on diffusion. CED utilises implanted intracranial microcatheters through which drugs are infused at precisely controlled infusion rates. The use of a specialised catheter and optimised infusion rate exploits bulk flow through the extracellular spaces of the brain [7]. CED is capable of homogeneously distributing drugs through large volumes of brain, independently of the size of the infused molecule [8]. Achieving maximimal distribution in a predictable and reproducable fashion by CED with minimal reflux of infusate is dependant upon a number of variables including catheter design, and the physicochemical properties and tissue affinity of the infusate [9] [10] [11]. Distribution is also dependant on infusate volume and time, as many infusates will ultimately be naturally cleared from the brain through innate interstitial fluid drainage pathways [12].

We have utilised CED of carboplatin to treat patients with recurrent/progressive glioblastoma and diffuse intrinsic pontine glioblastoma [13] on a compassionate treatment programme with an implantable catheter system incorporating a novel bone anchored transcutaneous port [14]. Carboplatin is hydrophilic, and as such is unable to diffuse freely across the blood—brain barrier. The direct intracranial administration by CED results in drug compartmentalisation within the brain, thereby reducing systemic toxicity. The potential of this treatment strategy to favourably impact the prognosis of patients with malignant brain tumours is likely to be dependent on achieving a consistent therapeutic drug concentration within a brain tumour and the surrounding penumbra [15] [16].

We sought to develop an alternative strategy to repeat dosing by encapsulating carboplatin in Poly(lactic-co-glycolic acid) (PLGA) copolymer, with the aim of achieving sustained release of the drug, thereby increasing the tissue half-life of carboplatin. Additionally, encapsulation may result in consistent exposure of the drug, thereby increasing tumour cytotoxicity. By delivering these nanoparticles by CED we also aim to reduce neurotoxicity.

PLGA was chosen as the US Federal Drug Agency (FDA) have approved its use in medical applications. It represents an ideal candidate for CED as it is biodegradable and hydrolyses into the natural metabolites lactic acid and glycolic acid. There have been no reports of safety concerns from the hydrolysed metabolites resulting from PLGA breakdown. In addition, the innate interstitial fluid drainage pathways would clear the acidic products from the brain. PLGA has previously been used to encapsulate paxlitaxel [17] and camptothecin [18] for treating glioblastoma by CED, highlighting its suitability for this application. Here we evaluate the potential of CED of carboplatin loaded PLGA nanoparticles as a novel treatment strategy for glioblastoma. We characterised the particles by their size and charge, followed by functional analysis of cytotoxicity in glioblastoma cell lines. The cell lines are invasive in nature [19] [20], and previous *in vitro* studies using these cell lines provided the basis for approval of a phase 1 clinical trial of CED of carboplatin for recurrent/progressive GBM [21] [22].

Using optimised conditions for CED into the striatum of rat brains [23], particles were analysed for their distribution, tissue retention and neurotoxicity. In order to further determine the translational potential of CED of carboplatin nanoparticles, we performed a neurotoxicity analysis in a large animal (porcine) model.

We demonstrate that carboplatin loaded PLGA nanoparticles offer substantial enhancements over free drug in terms of increased tumour cytotoxicity at lower infused concentrations, reduced neurotoxicity and increased tissue retention. This novel drug delivery strategy may have the potential to improve the prognosis for patients with glioblastomas.

## Methods

All reagents and animals used in this were commercially available. The animals used in this study were handled according to the protocols approved by the ethical committee of University of Bristol and all the protocols were performed in accordance with the UK Animal Scientific Procedures Act 1986.

### Synthesis of carboplatin PLGA-Nanoparticles (Carboplatin NP)

Carboplatin NP were produced using the double—emulsion method (W_1_/O_1_/W_2_). Briefly, 60 mg Poly (lactide-co-glicolide) (PLGA) (polymers with a 1:1 co-polymerization ratio ester ended (RG504 Mw 38-54KDa- Sigma-Aldrich, UK), acid ended (RG504H, Mw 38-54KDa—Sigma-Aldrich, UK) and poly(lactic acid) (PLA R203H Mw 18-24kDa- Sigma-Aldrich, UK) was dissolved in 2ml dichloromethane /ethyl acetate (DCM/EA) (both Sigma-Aldrich, UK) (2:8 V/V, O_1_ phase) and 0.7ml aqueous solution of carboplatin (10 mg/ml, W_1_ phase) (Accord Healthcare Limited, UK) was emulsified in the PLGA solution using a micro-tip probe sonicator (Model VC 600, Sonics & materials Inc., UK) set at level 4 for 3 minutes. Level 4 corresponded to 20 kHz at 45 W cm^-2^, and had been calibrated previously [24]. Encapsulation and release of aqueous components from sonochemically produced protein microspheres.

The primary (W_1_/O_1_) emulsion was transferred into 40 ml of Polyvinyl alcohol- Mw 20 KDa (PVA) (MP Biomedicals, USA). 2.5% solution (W_2_ phase) and the mixture was probe sonicated at level 4 for 5 minutes. The W_1_/O_1_/W_2_ emulsion was agitated by a magnetic stirrer uncovered overnight at room temperature to remove the organic solvent. In order to obtain particles with the desired diameter, the particle solution was treated by centrifugation (Centrifuge 5804R, Eppendorf, UK) at 9000 rpm for 15 minutes which caused the large particles to form a pellet while the smaller particles remained in the supernatant. The pellet of large nanoparticles was discarded while nanoparticles in the supernatant were collected and washed by ultracentrifugation (40000rpm for 20 minutes, Motor type 70Ti/70.1Ti, L-80 ultracentrifuge, Beckman Coulter, UK). This pellet of nanoparticles was re-suspended in water, freeze-dried and stored at -20°C for further usage. For fluorescent carboplatin nanoparticles (fluorescein carboplatin NP), PLGA was substituted with the mixture of PLGA (Mw 38-54KDa) and PLGA-Fluorescein end cap copolymer (Mw 7KDa and 30K-40K, purchased from Polyscitech, IKINA, USA) with the weight ratio of 9:1, and processed as described.

### Nanoparticle characterisation

Freeze-dried nanoparticles were completely re-suspended in aqueous solution by an ultrasonic bath (Fisherbrand FB 11020) for 15 minutes. A Zetasizer-nano (Malvern Instruments, Worcestershire, UK) using light from a He-Ne laser source (λ = 633nm) was used. Dynamic light scattering measurements were taken to generate particle size and polydispersity values. All experiments were performed at 25°C at a scattering angle of 173° to the incident beam, with an assumed refractive index ratio of 1.59 and viscosity of 0.89 cP. The correlation decay functions were analysed by the cumulants method to obtain an average hydrodynamic particle size and polydispersity. The intensity-weighted mean value presented is the mean of three measurements. ζ-potential was also determined with the Zetasizer nano ZS. By measuring the electrophoretic mobility, the ‘zeta’—potential can be calculated using Henry equation within the Smoluchowsky approximation.

Morphological analysis of the PLGA nanoparticles was performed using TEM (JEOL JEM 1200 EXII). A drop of NP suspension was placed on a Formvar (poly(vinylformal)/carbon supported 400 mesh grid, excess liquid was removed with a filter paper, and the grid was dried in air at room temperature followed by negatively stained with phosphotungstic acid solution (3% W/V). TEM images were calibrated using SIRA gratings 2160 (462nm) lines per millimeter and catalase crystals for high magnification (8.75/6.85nm spacing) in the unit cell.

### Quantification of carboplatin loading

Carboplatin NP (5mg) were suspended in 0.5ml artificial Cerebral Spinal Fluid (aCSF) and placed in a centrifuge filter tube (MCOFF 10 K) in a 37°C oven. At defined periods, samples were centrifuged at 11000 rpm for 15 minutes with the aCSF solution collected and replaced with fresh aCSF. Finally, the remaining nanoparticles were collected and dissolved in 0.6ml DCM and vortexed for 3 minutes. 1ml aCSF was added to the solution and the mixed solution was then vortexed for 3 minutes to extract carboplatin into the aCSF solution. This solution was then centrifuged at 11000 rpm for 15 minutes, and the upper aqueous solution containing the extracted carboplatin was removed by pipette and collected before fresh aCSF solution was replaced onto the DCM solution. The whole extraction process was repeated five times to make sure that any remaining carboplatin in the particles was fully extracted. The carboplatin concentration in the collected fractions was measured by Inductively Coupled Plasma Mass Spectrometry (Thermo Fisher Scientific XSeries 2 ICP-MS) at the Southampton Oceanography Center, UK. Iridium (Ir) was used as internal standard due to its similar mass and ionization potential. Quantification was determined against a calibration curve using weighted linear regression analysis.

### Cell culture and primary culture of hippocampal neurones and glial cells

Invasive GBM cancer cell lines [25] were cultured in Dulbeccos modified Eagle medium (DMEM; Life Technologies, Rockville, MD, USA) supplemented with foetal calf serum (FCS; Life Technologies, Rockville, MD, USA), L-glutamine and penicillin/streptomycin solution (both Sigma Aldrich, Dorset, UK). Neuronal cultures were obtained from the hippocampus of 18 day old Wistar rat embryos. Briefly, brains were removed and freed from meninges, and the hippocampus isolated in Hank’s Balanced Salt solution (HBSS; Life Technologies, Rockville, MD, USA). Cells were then dispersed by incubation for 20 minutes at 37°C in a trypsin solution followed by mechanical trituration. The cell suspension was diluted in Neurobasal media (NB; Life Technologies, Rockville, MD, USA) supplemented with 1x B27, 2% Foetal Calf Serum (both Life Technologies, Rockville MD, USA), penicillin/streptomycin and L-glutamine. These were counted and plated on poly-D-lysine (all Sigma Aldrich, Dorset, UK) coated coverslips, or poly-D-lysine coated 24 well plates at a density of 75000 cells per coverslip or well. Half of the medium was replaced 3 days later. Experimentation on these cells was conducted after another 2 days.

### *In vitro* cytotoxicity assay

Cellular cytotoxicity was measured using the MTT (3-(4,5-dimethylthiazol-2-yl)-2,5-diphenyltetrazolium bromide) colorimetric method, which uses the conversion of MTT by the intact mitochondria of living cells to a coloured product, formazan, the concentration of which was measured by a spectrophotometer. Following defined periods of culture, cells were dosed with either carboplatin (hippocampal and glial cell culture; 0.03mg/ml, UPAB; 0.18mg/ml and SNB19; 0.03mg/ml) or carboplatin NP at a concentration of 1 mg/ml for up to 72 hours prior to the addition of MTT. Following a 3 hour incubation of MTT at 37°C, the cell culture media was discarded and acidified isopropanol was added. After slight agitation the absorbance was measured by a spectrophotometry at 450nm. All assays were performed in triplicate on 3 separate occasions. Experimental values were normalised against the negative control.

### *In vitro* nanoparticle uptake analysis

Following defined periods of culture onto glass coverslips, cells were dosed with 1mg/ml fluorescein carboplatin NP. After defined periods, cells were fixed with 4% paraformaldehyde for 20 minutes and washed with PBS. Coverslips were subjected to immunofluorescent procedures and mounted with Vectashield with 4',6-diamidino-2-phenylindole (DAPI; Vector Laboratories Inc., Burlingame, CA). Coverslips were labelled with Alexa Fluor 568 phalloidin (Invitrogen Life Technologies) for 20 minutes at room temperature.

### *In vitro* neurotoxicity assessment

Primary hippocampal cells were processed and plated onto glass coverslips until neuronal connections were visible. Following the *in vitro* dosing regimen, coverslips were fixed with 4% paraformaldehyde for 20 minutes and washed with PBS. Subsequently, coverslips were subjected to standard immunofluorescent protocols, and mounted with Vectashield with DAPI. The coverslips were incubated with either anti-B3tubulin (1:2000; Millipore, CA, USA) or rabbit anti-GFAP (1:300; Millipore, CA, USA) primary antibodies overnight at 4°C. Detection was determined using goat anti-mouse Alexa Fluor 488 for B3tubulin or goat anti-rabbit Alexa Fluor 488 for GFAP (both Invitrogen Life Technologies).

### Statistical Analysis

For statistical analysis IBM SPSS 21 program (New York, USA) was used. Paired *t-test* was utilised with significance set at p = 0.05.

### *In vivo* Convection Enhanced Delivery (CED)—small animal model

Adult male Wistar rats (Charles River, Margate, UK, 225 to 275g) were anaesthetised with intraperitoneal ketamine (Ketaset; 60mg/kg, Pfizer Animal Health, Sandwich, UK) and medetomidine (Dormitor; 0.4mg/kg, Pfizer. USA), and then placed in a stereotactic frame (Stoelting, Illinois, USA). A midline skin incision was made from glabella to occiput to expose bregma. Bilateral burr holes were drilled using a 2mm drill. CED procedures were performed using a custom-made catheter with an outer diameter of 0.22mm and inner diameter of 0.15mm, composed of fused silica with a laser cut tip. The cannula was attached to a 1ml syringe (Hamilton, Bonaduz, Switzerland) connected to a rate-controlled microinfusion pump (World Precision Instruments Inc., Sarasota, FL, USA) and the tip placed at stereotactic co-ordinates derived from the Paxinos and Watson stereotactic rat brain atlas (0.75mm rostral and 3mm lateral to bregma, depth 5.0mm in order to target the striatum [26]. All CED procedures were performed at an infusion rate of 1μl/minute. 10μl of carboplatin NP (1mg/ml) or fluorescein carboplatin NP (1mg/ml) in aCSF was infused into the striatum. On completion of CED the cannula was left in situ for 10 min to minimise reflux, then withdrawn at a rate of 1mm/minute. The wound was closed with 4/0 Vicryl, and a dose of intramuscular buprenorphine (Centaur Services, Castle Cary, UK) was administered (30μg/kg). The anaesthetic was reversed with 0.1mg/kg i.p. atipamezole hydrochloride (Pfizer) in recovery procedures. Rats were euthanised by anaesthetic overdose with an intraperitoneal injection of 1ml pentobarbital (Euthatal; Merial Animal Health, Harlow, UK) at pre-defined time-points following CED of aCSF (control), carboplatin or carboplatin NP (2 weeks or 4 weeks).

### *In vivo* nanoparticle distribution analysis—small animal model

Following CED of fluorescent carboplatin NP (1mg/ml; 5μl volume) into the striatum, Brains were removed and placed in 4% paraformaldehyde for 24 hours, then cryoprotected in 30% sucrose. Rat brains were then cut into 35μm thick coronal sections using a Leica CM1850 cryostat (Leica Microsystems, Wetzlar, Germany) at -20°C. Sections were mounted on gelatine-subbed slides and subsequently counterstained with DAPI.

### *In vivo* tissue retention analysis—small animal model

To analyse the tissue retention of carboplatin or carboplatin NP, brains were explanted and samples taken around the infusion point using a 3mm diameter sample corer. Samples were thawed and homogenised twice in aCSF at 5000rpm for 20 seconds using a Precellys 24 homogenizer (Bertin Technologies, USA). Tissue homogenates were subsequently diluted with water or nitric acid (2%, v/v) and analysed by ICP-MS detection using the Hydrogen mode (Agilent Technologies 7700 series) equipped with an autosampler (Agilent G3160B) for platinum content. All time points were carried out in triplicate with experimental values normalised against 0 hours control.

### *In vivo toxicity*—small animal model

For immunohistochemical analysis (IHC), animals were transcardially perfused with 4% paraformaldehyde. Brains were removed and placed in 4% paraformaldehyde for 24 hours, then cryoprotected in 30% sucrose. Rat brains were then cut into 35μm thick coronal sections using a Leica CM1850 cryostat (Leica Microsystems, Wetzlar, Germany) at -20°C. Sections were mounted on gelatine-subbed slides and subsequently subjected to standard immunofluorescent protocols in order to identify neuronal disruption and gliosis.

The sections were incubated with either mouse anti-NeuN (1:100; Millipore, CA, USA) or rabbit anti-GFAP (1:300; Millipore, CA, USA) primary antibodies overnight at 4°C. Detection was determined using Donkey Anti-Mouse (1:150; streptavidin Alexa Fluor 488 Jackson Laboratories, Sacramento, CA, USA) or donkey anti-rabbit Cy3 (1:300; Jackson Laboratories, Sacramento, CA, USA). Sections were also treated with DAPI (1:200 of 1mg/mL; Sigma Aldrich, Dorset, UK) prior to mounting in Fluorsave Reagent (Calbiochem, Merck Millipore, Billerica, MA, USA). Images were captured using the Stereo Investigator platform (MicroBrightField Bioscience, Williston, VT, USA) with a Leica DM5500 microscope (Leica Microsystems, Germany) and digital camera (Microbrightfield Bioscience).

### *In vivo* convection enhanced delivery (CED)—large animal model

A total of three large white pigs (40–45kg) were used in order to assess toxicity in this study. Pre-anaesthetic medication comprising azaparone (2 mg/kg, Janssen Ltd., Bucks, UK) and ketamine (10 mg/kg, Vetoquinol Ltd. Buckingham, UK) were administered by deep intramuscular injection into the dorso-lateral neck muscles. Propofol (Abbot Laboratories (Kent, UK) was used for induction of anaesthesia, maintained with isofluorane (Isoflo, Abbot Laboratories). Morphine 0.1 mg/kg (Morphine sulphate, Martindale Pharmaceuticals Ltd., Essex, UK) and Meloxicam 0.4 mg/kg slow IV, (Metacam 20 mg/ml solution for injection, Boehringer Ingelheim Vet Medica GmbH., Ingelheim, Germany) were administered for analgesia by intravenous injection. Head immobilisation and brain imaging were achieved as previously described [14–16]

### Nanoparticle infusion regimen—large animal model

Carboplatin NP were diluted in sterile aCSF (Torbay Pharmaceutical Manufacturing Unit, Paignton, UK) in combination with 0.2% Gadolinium-DTPA (Magnevist, Bayer Healthcare, Germany). Carboplatin NP were infused into the left putamen, and aCSF alone as a control into the right putamen using the following ramping regime: 0.5 μl/min for 5 min, 1.0 μl/min for 5 min, 2.5 μl/min for 5 min, 5 μl/min for 10 min ± 10 μl/min until completion.

Imaging was undertaken using an MRI scanner with field strength of 1.5T (Intera, Philips, UK). Pre-operative MR imaging comprised contiguous T1-weighted coronal slices (0.8 mm slice thickness) of a volume which included fiducials and brain. For subsequent T1-weighted imaging (repeated at 15 minute intervals) the parameters were—FOV: AP 200mm: RL 159 mm: FH72 mm; voxel size: AP 0.575mm: FH 0.8 mm; matrix size: M x P 378 x 277. The brain was aligned on the anterior commissure posterior commissure line (AC-PC) to facilitate comparison with histological analysis. After the first infusion (1mg/ml), a subsequent two infusions were carried out at 2 mg/ml and 3 mg/ml to achieve a dose escalation. The total volume of infusion ranged from 60μl to 120μl per catheter. A total of 9 infusions performed in 3 animals were used for toxicity analysis and volume distribution. Animals were terminated one month after the third successful delivery of Carboplatin NP or aCSF alone via transcardial perfusion-fixation with 10% formalin. Brains were explanted and post-fixed in 10% formalin for 2 weeks, then cryprotected in 30% sucrose-formalin.

### *In vivo toxicity*—large animal model

Pig brains were aligned on AC-PC prior to sectioning to facilitate comparison with MR images, and cut into 100 μm coronal sections using a Leica SM2500 microtome (Leica microsystems, Milton Keynes, UK). In order to assess the toxicity, 6 sections anterior and posterior to the catheter track were assessed against the control infusions of aCSF. Sections were mounted on gelatine-subbed slides and subsequently subjected to standard immunofluorescent protocols in order to in order to identify neuronal disruption, gliosis and increased macrophage activation.

The sections were incubated overnight at 4°C with either mouse anti-NeuN (1:100; Millipore, CA, USA), rabbit anti-GFAP (1:300; Millipore, CA, USA) or CD63 (1:100; Serotec, Oxfrod UK). Detection was determined using Donkey Anti-Mouse (1:150; streptavidin Alexa Fluor 488) or donkey anti-rabbit Cy3 (1:300; Jackson Laboratories, Sacramento, CA, USA) or Donkey Anti-Mouset Alexa Fluor 488 (1:300, Jackson Laboratories, Sacramento, CA, USA). Sections were also treated with DAPI (1:200 of 1mg/mL; Sigma Aldrich, Dorset, UK) prior to mounting in Fluorsave Reagent before visualisation. Images were captured as described earlier.

## Results

### Development and characterisation of carboplatin NP

A number of nanoparticle formulations were manufactured and tested *in vitro* in order to achieve the optimal preparation for progression to *in vivo* testing. We utilised an iterative approach to nanoparticle formulation, making stepwise alterations to the ratio of polymer components, solvents and synthesis methods. The double emulsion (Water/Oil/Water) method was selected for progression to *in vivo* testing because it consistently yielded nanoparticles whose size and surface charge were suitable for CED with minimal nanoparticle aggregation.

**Fig 1:**
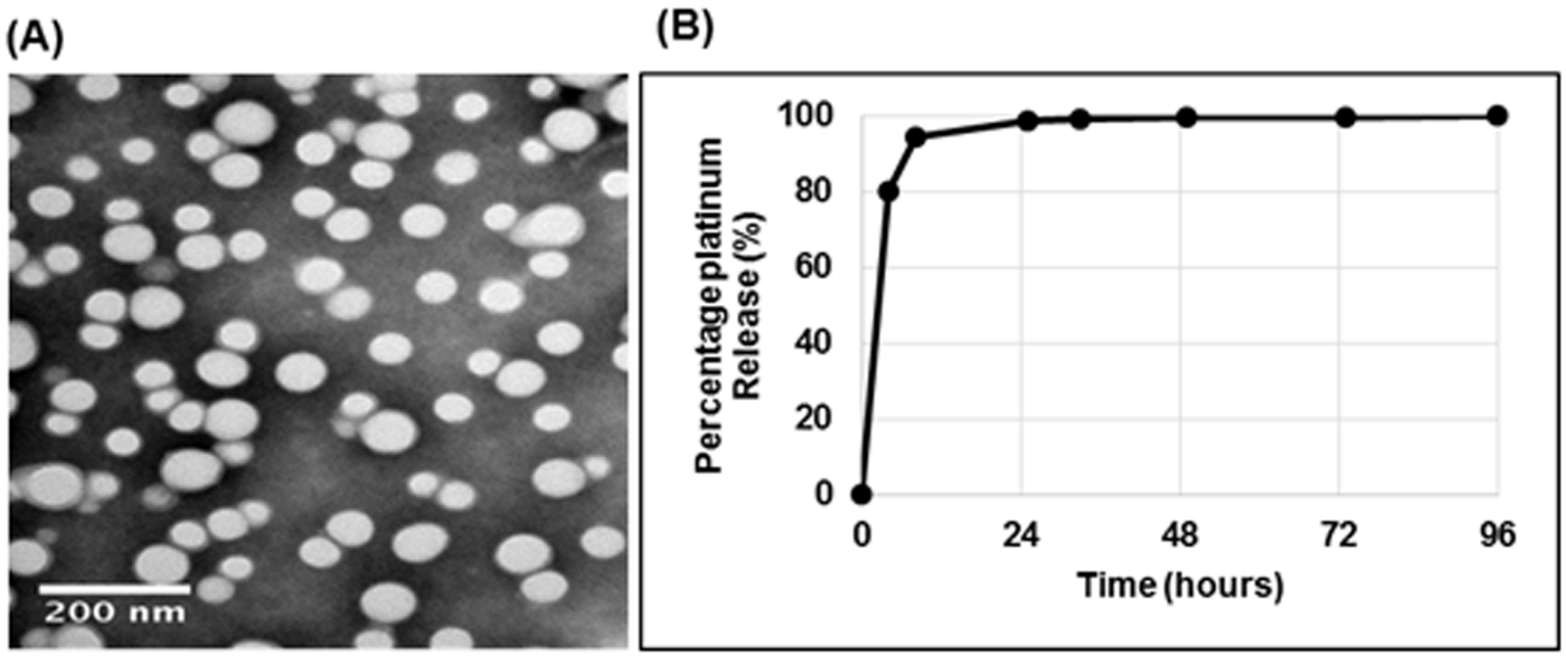
Characterisation of carboplatin NP. (A) Carboplatin NP show a consistent spherical shape, less than 200nm in size. Morphological analysis of the nanoparticles was conducted using TEM scaled at 200nm. (B) Approximately 100% of carboplatin is released within 24 hours. Release profile of carboplatin from nanoparticles was conducted by analysing platinum content by ICP-MS at defined timepoints.

The double emulsion method yielded nanoparticles exhibiting a smooth spherical shape with an average diameter of 175nm ± 40.75nm and a surface charge of -31.18 ± 7.31mV. The percentage loading of carboplatin in these particles as determined by ICP-MS was 0.27%. TEM analysis showed particles no more than 200nm in size, without aggregation (Fig 1A). In aCSF solution, the particles dispersed extremely well, and remained stable for 2 months (data not shown). Analysis of platinum release from the nanoparticles revealed 100% release after 24 hours (Fig 1B).

### Carboplatin NP have increased cytotoxic effect against glioblastoma cancer cells

**Fig 2:**
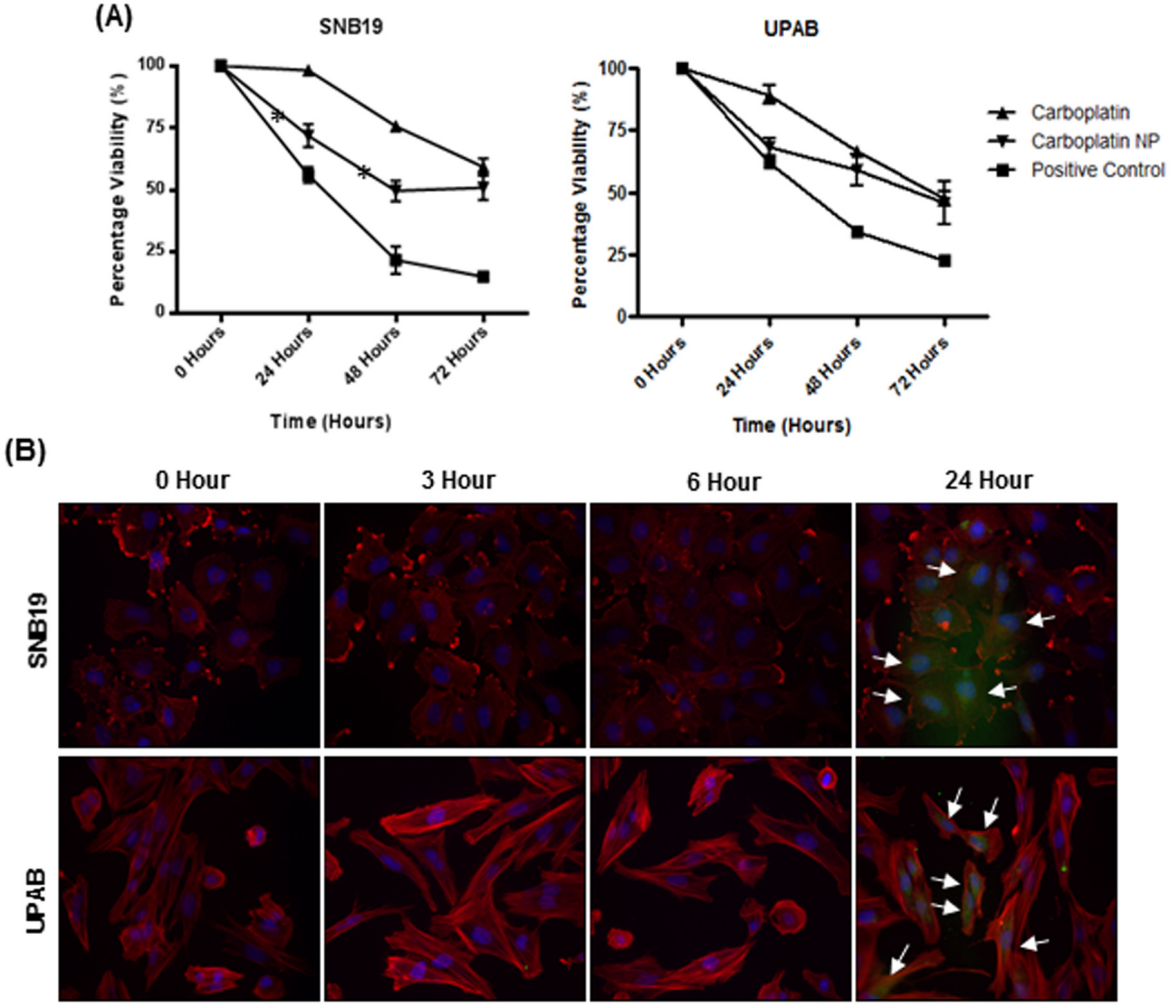
*In vitro* cytotoxicity and uptake of carboplatin NP. (A) Carboplatin NP showed increased cytotoxicity over 48 hours. The cytotoxic effect of the carboplatin nanoparticles (Carboplatin NP) were assessed by MTT assay in UPAB and SNB19 human glioblastoma multiforme (GBM) cell lines. For UPAB, 0.18mg/ml and for SNB19, 0.03mg/ml carboplatin was used as these concentrations represent the IC50 after 72 hours. Paired *t-test* statistical analysis comparing cytotoxicity revealed significant differences for SNB19 at 24hours (p = 0.001) and 48 hours (p = 0.004), indicated by asterix. (B) Uptake of fluorescein-labelled carboplatin NP (green) occurs within 24 hours of dosing. Cells were dosed and fixed after defined periods of culture. Cells were stained with phalloidin (red), to visualise actin cytoskeleton and DAPI (blue) for cell nuclei. Uptake into cells is indicated by white arrows.

The cytotoxic effect of carboplatin NP was compared to free carboplatin in two human glioblastoma cell lines (UPAB and SNB19). Cells were dosed with either 1mg/ml of carboplatin NP (with carboplatin loading of 0.27%) or carboplatin, 0.18 mg/ml for UPAB and 0.03 mg/ml for SNB19. These concentrations of carboplatin were selected as they represent the IC50 at 72 hours for each cell line. Dosing with carboplatin NP resulted in a statistically significant increase in cytotoxicity at 24 and 48 hours in SNB19 cultures (p = 0.001 and p = 0.004, paired t-test). There was a non-significant trend towards increased cytotoxicity with carboplatin NP in UPAB culture at 24 and 48 hours. At 72 hours the cytotoxic effect of carboplatin NP was comparable to that of the carboplatin alone for both cell lines (Fig 2A). The cellular uptake of a comparable fluorescent NP in GBM cells occurred within 24 hours. (Fig 2B).

### Encapsulation of Carboplatin in PLGA NP confers a reduction in neurotoxicity *in vitro*

**Fig 3:**
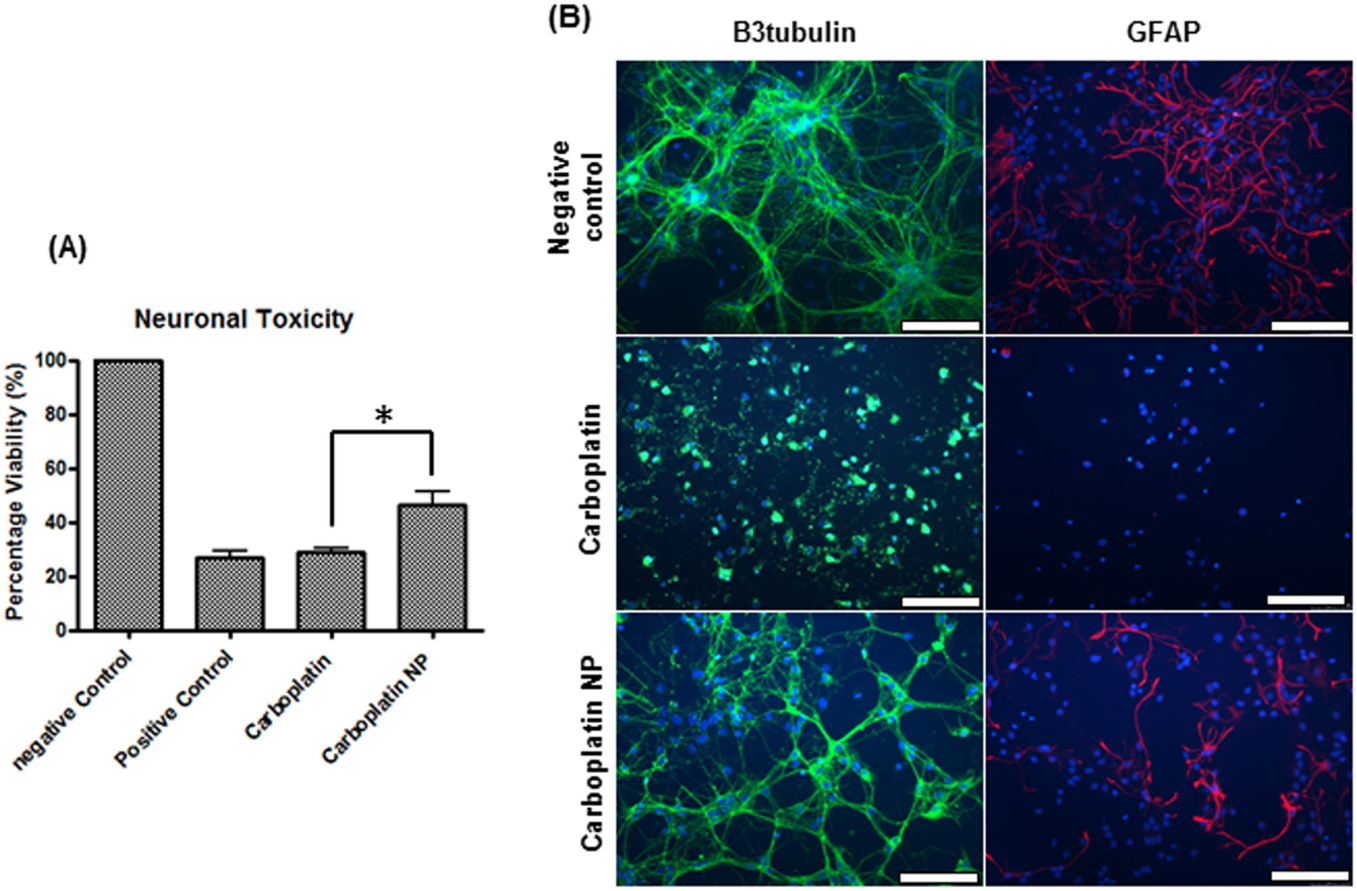
*In vitro* neurotoxicity in primary rat hippocampal cultures. Carboplatin NP are less toxic to neurones compared to the free drug. Primary rat brain hippocampal cultures were dosed with either carboplatin (0.03mg/ml) or carboplatin NP (1mg/ml) and assayed after 72 hours of culture. (A) MTT analysis shows significant increase in cell viability with carboplatin NP (p<0.001). (B) Carboplatin alone causes deregulation of the neurones and loss of the glial cells, whilst carboplatin NP retained neuronal connections and glial cells. Immunofluorescent analysis of the neurones (B3tubulin; green) and glial cells (GFAP; red). Cells were counterstained with DAPI to visualise cell nuclei (blue) (Scale bar; 100μm).

A significant reduction in neurotoxicity was seen after dosing a mixed primary culture with carboplatin nanoparticles compared with carboplatin alone (p<0.001, Fig 3A). Immunofluorescence assessment of B3tubulin, a widely used marker for neuronal integrity, and GFAP, a marker for glial cells revealed that carboplatin alone causes complete deregulation of the neurones and loss of glial cells after 72 hours of dosing. Although a degree of toxicity was observed with carboplatin NP, morphologically the neurones were intact and GFAP labelling showed preservation of glial cells (Fig 3B).

### CED efficiently distributes Carboplatin NP within a small animal model

**Fig 4:**
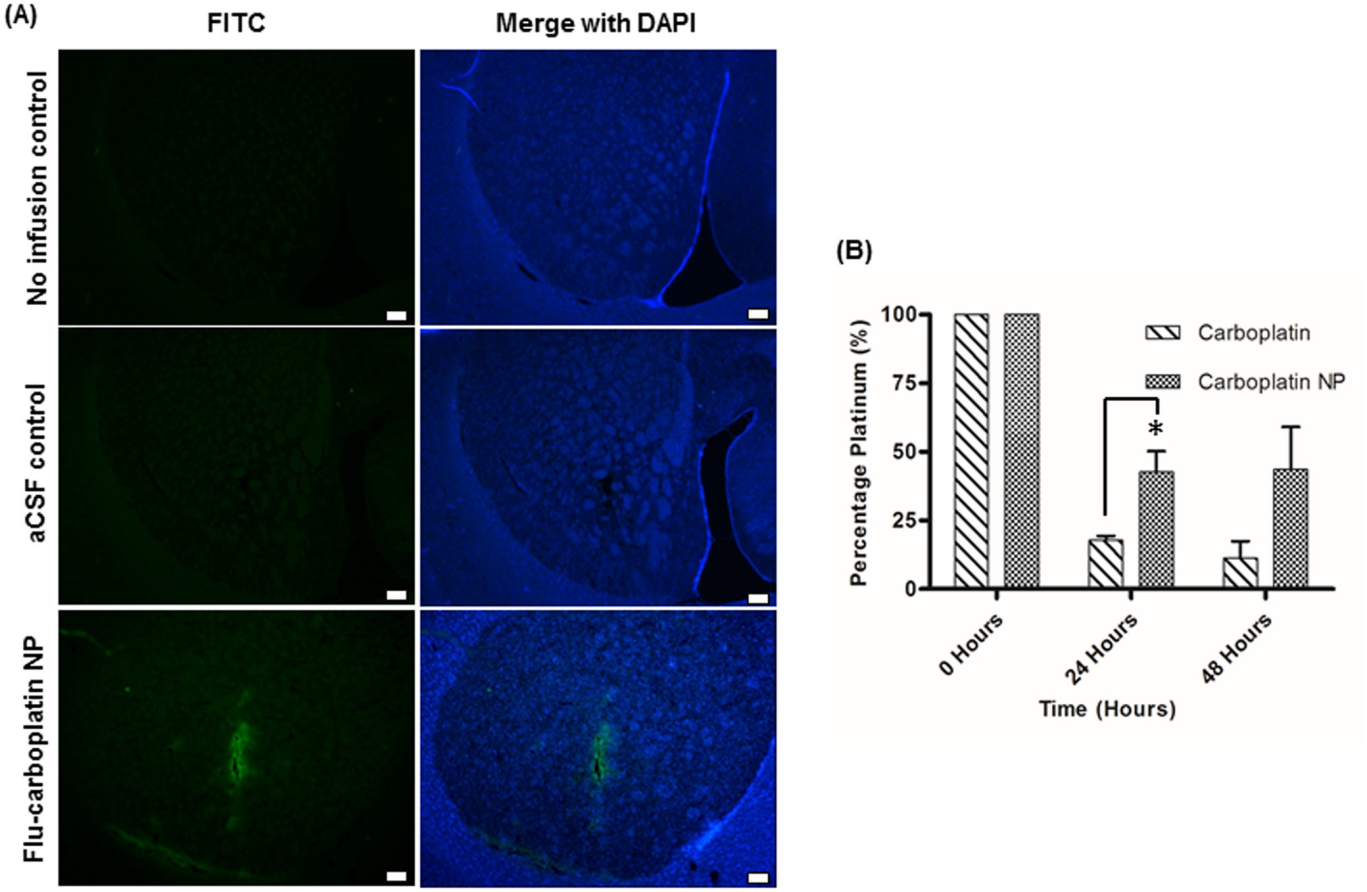
CED of carboplatin NP into the striatum of rat brains. Immediately after CED, nanoparticles distribute throughout the striatum. (A) CED of aCSF alone (5μl volume) or fluorescein-labelled carboplatin NP into the striatum of rat brain was conducted (1mg/ml; 5μl volume). These were analysed by sectioning around the needle track and counterstaining with DAPI (Scale bar; 250μm). (B) Carboplatin NP show increased tissue retention after 24 and 48 hours. Following CED of carboplatin NP (1mg/ml; 5μl volume), punches of the brain tissue obtained around the site of infusion were analysed by ICP-MS. Paired *t-test* analysis revealed a significant increase in the tissue half-life of carboplatin NP at 24 hours (indicated by asterix).

A comparative PLGA carboplatin nanoparticle incorporating fluorcein was utilised to analyse the distribution of carboplatin NP. This was infused into the striatum of a rat brain, and the animal sacrificed immediately after the infusion. Immunofluorescent imaging of striatal tissue sections at 488nm revealed distribution of NP throughout the striatum (Fig 4A).

### Encapsulation of carboplatin in PLGA enhances tissue retention *in vivo*

Carboplatin NP clearance was examined *in vivo*, in order to assess the nanoparticles’ ability to achieve sustained release over time. Rat brains were infused with either carboplatin or carboplatin NP, and tissue punches around the needle track were obtained after given periods of time. This was analysed for platinum content by ICP-MS to give a measure of clearance. Comparison of carboplatin with carboplatin NP showed that the nanoparticles were retained within the tissue for a significantly greater period of time (p = 0.03, Fig 4B).

### Carboplatin NP show no signs of neurotoxicity *in vivo*

**Fig 5:**
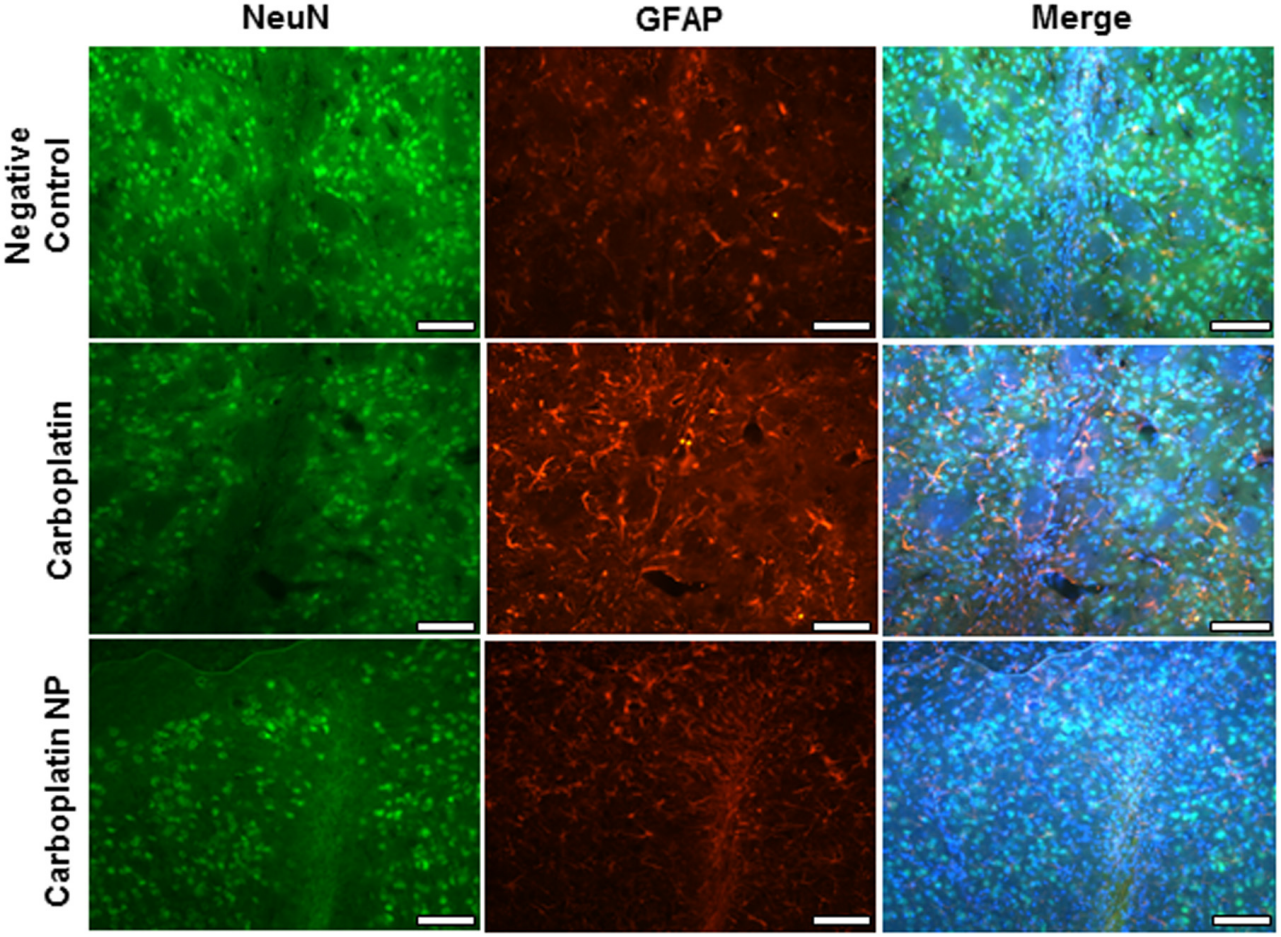
Toxicity analysis after CED into the striatum of rat brains. CED of aCSF (negative control), carboplatin (0.72mg/ml; 5μl volume) (or carboplatin NP (1mg/ml; 5μl volume) was conducted and toxicity in rat striatum assessed. Dual IHC analysis of neurons (NeuN) and Glial cell (GFAP) demonstrated minimal toxicity localised to the needle track. No glial or neuronal cell loss was observed elsewhere. Scale bar: 100μm.

Infusions of aCSF (negative control), carboplatin or carboplatin NP were conducted into the striatum of rat brains by CED. No gross morphological changes were observed following CED of aCSF, carboplatin or carboplatin NP. Minimal neuronal and glial disruption was observed in relation to the needle track and was comparable for all 3 study groups (Fig 5).

### CED efficiently distributes carboplatin NP within a large animal model

**Fig 6:**
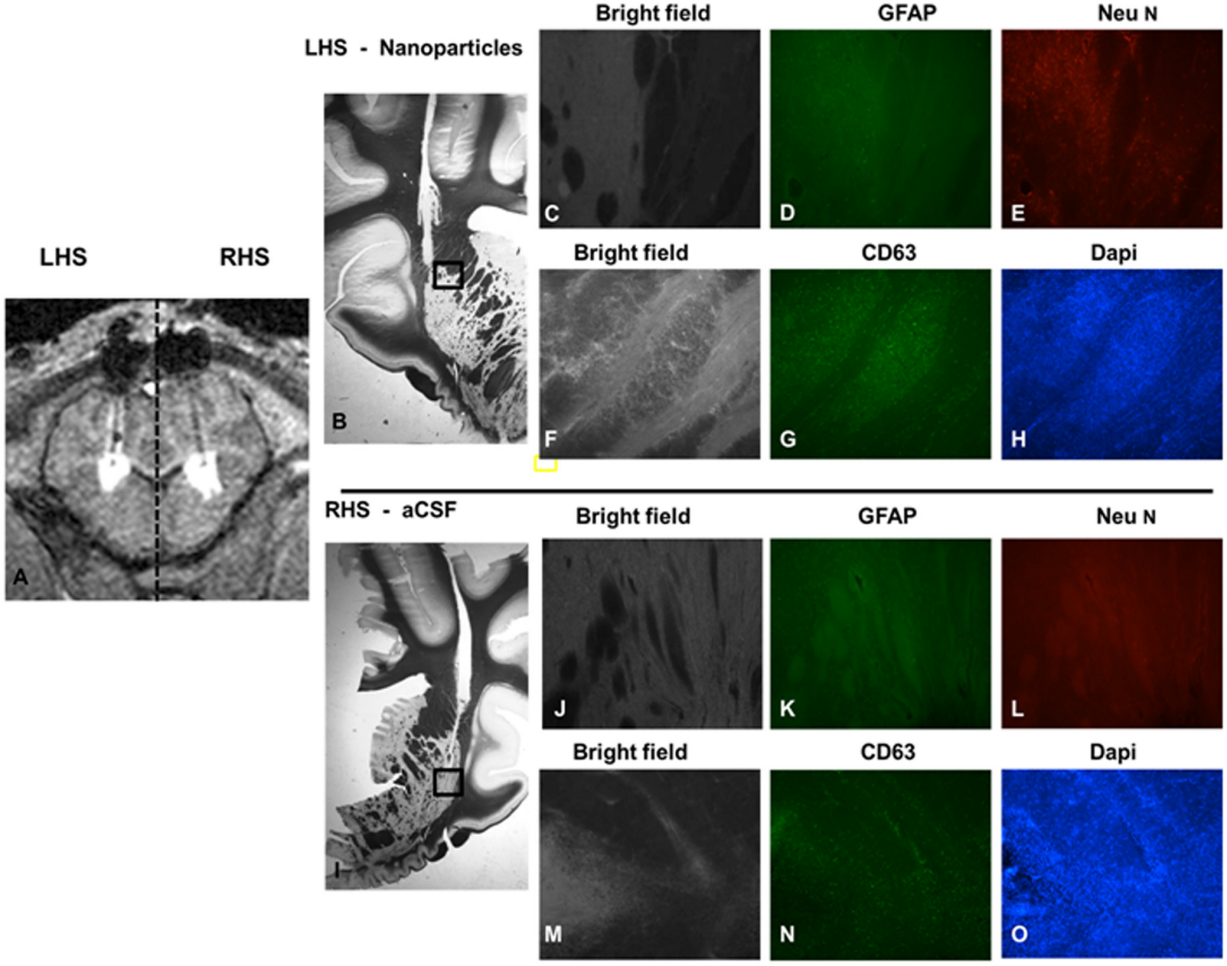
Toxicity assessment after dose-escalation study in pigs. A dose-escalation study comparing CED of carboplatin NP with aCSF (co-infused with 0.2% Gd) was conducted in a porcine model. (A) Widespread putamenal distribution was observed using real-time MR imaging (B) Fluorescence immunohistochemistry demonstrates intact neuronal (E & L) and glial (D & K) networks at the site of infusion. Repeat dosing of carboplatin NP was not associated with increased macrophage activation compared to control (G & N). Minimal toxicity was also confirmed by maintenance of cellular architecture (H & O) (Scale bar; 250μm in magnified images, 100μm in images showing entire section (B&I)

Intermittent intraputamenal infusions of carboplatin NP or control infusions of aCSF were successfully performed over a three month period. All catheters were accurately inserted to target and achieved satisfactory putamenal distribution based in T1 weighted MRI (Fig 6A). No animals demonstrated abnormal behaviours or weight loss throughout the study period. Serial blood tests did not show any adverse effect on haematological parameters attributable to carboplatin NP throughout the experimental period.

### CED of carboplatin NP is not associated with toxicity in a large animal model

After 1 month post infusion of carboplatin NP, there were no adverse haematological or behavioural effects. There were no gross morphological changes were observed within the putamen following CED of aCSF or carboplatin NP, with the exception of the catheter track (Fig 6C, 6F, 6J and 6M). The porcine dose escalation study did not reveal any immunohistochemical evidence of neuronal loss, gliosis or adverse immune response attributable to infusion of carboplatin NP (Fig 6D, 6E, 6G, 6H, 6K, 6L, 6N and 6O).

## Discussion

Despite intense research, the prognosis for patients with glioblastoma remains poor. Patients eligible for surgery, radiotherapy and chemotherapy have a median survival of less than 15 months [27]. There is an urgent need to develop more effective treatment strategies for this devastating condition.

One of the major barriers to the effective treatment of glioblastoma is the BBB which prevents the free passage of chemotherapies from the bloodstream into the brain [3,28]. Convection-enhanced delivery has emerged as a promising strategy for bypassing the BBB, and allows anatomically targeted delivery of chemotherapeutic agents to a tumour and the surrounding brain tissue [29,30]. Our research group has focussed on the clinical translation of CED of carboplatin for glioblastoma [14,21,31]. Our experience suggests that the potential of this treatment strategy to favourably impact the prognosis of patients with malignant brain tumours is likely to be dependent on achieving a consistent therapeutic drug concentration within a brain tumour and the surrounding penumbra. In this study, we aimed to determine whether carboplatin encapsulated in PLGA could offer advantages over the delivery of free carboplatin by improving cytotoxicity, reducing neurotoxicity and increasing tissue half-life.

To elucidate these aims, carboplatin NP were synthesised and their size and charge characterised. The particle formulation selected for *in vivo* studies were <200nm in diameter with a negative charge, as we previously determined that nanoparticles greater than 200nm do not distribute effectively when delivered by CED (unpublished data). Previous studies investigating the effects of particle charge on CED together with our own pilot studies have confirmed that optimum distribution is seen with negatively charged particles of -20 to -50mV [10]. Unfortunately, the release profile of carboplatin from the PLGA nanoparticles occurred within 24 hours. We attribute this to the drug being absorbed into the nanoparticle surface. Indeed, it known that hydrophilic drugs suffer from low loading and a burst release profile when encapsulated with PLGA [32]. However, the particle may be modified by addition of poly(methyl methacrylate-co-methacrylic acid) (PMMA—MA) and adjustment of the aqueous phase pH which may increase drug loading and maintain a delayed release profile [33].

Despite the burst release and low loading efficiency of the carboplatin NP, dosing of human glioblastoma cell lines resulted in improved cytotoxicity compared to free carboplatin over 48 hours. Further investigation is required determine the number of particles required to achieve sufficient efficacy in clinic. However, since GBMs are heterogeneous in nature, the number of particles required may vary depending on tumour environment.

We have shown uptake by GBM cells within 24 hours with a comparable fluorescein-labelled NP. The improvement of cytotoxicity due to cellular uptake of nanoparticles has been previously reported in a number of tumour types, including glioblastoma models [10,34,35]. We cannot discount that the addition of fluorescein may have altered the NP formulation. As the nanoparticle does not contain other markers amenable to analysis, this remains the best viable option to demonstrate distribution and cellular uptake.

The neurotoxic effects from the nanoparticles were assessed *in vitro*. Our clinical experience has shown that free carboplatin remains around the infusion site for ~72 hours (unpublished data). To represent the clinical situation, the *in vitro* neurotoxic analysis was assayed over 72 hours. *In vitro*, carboplatin NP maintained a degree of glial and neuronal integrity compared to carboplatin alone. However, *in vivo* we did not observe any significant neuronal or glial cell loss with either carboplatin or carboplatin NP. We speculate that an acute infusion by CED coupled with clearance from the rat brain would supersede a cellular neurotoxic response.

The porcine model offers advantages over small animals as the technology of infusing small animals by CED currently does not allow for repeat infusions over a longer period of time with a vast increase the volume of infusate. Utilising the porcine model, a thorough assessment of neurotoxicity was conducted through the observation of behaviour, blood testing and immunohistochemical analysis one month post infusion of the nanoparticles. No adverse results seen in terms of behaviour, haematological changes or immune response. Taken together, this highlights that the carboplatin NP would be safe to use in clinic. We recognise that an extensive evaluation of neurotoxicity by assessment of motor activity, cognition, motor and sensory activities may be required for further translation.

A desirable quality to have in the clinic would be to increase the tissue half-life of the chemotherapeutic. This would allow for reduced frequency of dosing in the clinic, thereby reducing the number of hospital attendances required by the patient. To investigate this, following CED into the striatum of the rat brain, the kinetics of the nanoparticles were analysed by ICP-MS for platinum content. Despite the burst release profile of the nanoparticles, after 24 hours there was an increase of 25% platinum of the carboplatin NP treatment compared to carboplatin alone. This confirmed improved tissue retention of the carboplatin NP over time and therefore prolonged tissue half-life. While the results presented show promise, further study is required to determine the translational potential of this novel drug delivery strategy. Although an *in vivo* tumour model may be useful for further pre-clinical investigational work, our treatment strategy in the clinic is not to target tumours with a large solid component which may be more amenable to resection, but rather to target the peritumoral sites of tumour recurrence [14]. This strategy negates the need for a *in vivo* tumour model as this would not recapitulate the biology of the clinical tumour and the treatment regime which includes tumour resection.

We also recognise that a thorough assessment of the volume of distribution:volume of infusion is required to fully investigate how infiltrative the nanoparticles are. Further modification of the particle may be interesting avenue to explore, as addition of poly(ethylene) glycol (PEG) has been shown to aid the penetrating properties of the nanoparticle [36]. Further improvements would include incorporating a contrast agent to aid magnetic resonance imaging, which is readily utilised within the clinic.

To the best of our knowledge this is the first report of the possible application of carboplatin/PLGA nanoparticles as a potential treatment of glioblastoma by CED. We have successfully generated carboplatin loaded PLGA nanoparticles which show advantages over the use of carboplatin alone. Our findings suggest that this novel drug delivery system with further modification and clinical investigation may in the future have the potential to improve the prognosis for patients with glioblastomas.

## References

[1] Stupp R, Mason WP, van den Bent MJ, Weller M, Fisher B, Taphoorn MJ, et al Radiotherapy plus concomitant and adjuvant temozolomide for glioblastoma. The New England journal of medicine. 2005;352(10):987–96. 15758009 10.1056/NEJMoa043330

[2] Wallner KE, Galicich JH, Krol G, Arbit E, Malkin MG. Patterns of failure following treatment for glioblastoma multiforme and anaplastic astrocytoma. International journal of radiation oncology, biology, physics. 1989;16(6):1405–9. 10.1016/0360-3016(89)90941-3 2542195

[3] Whittle IR, Malcolm G, Jodrell DI, Reid M. Platinum distribution in malignant glioma following intraoperative intravenous infusion of carboplatin. British journal of neurosurgery. 1999;13(2):132–7. 10616580 10.1080/02688699943871

[4] Westphal M, Hilt DC, Bortey E, Delavault P, Olivares R, Warnke PC, et al A phase 3 trial of local chemotherapy with biodegradable carmustine (BCNU) wafers (Gliadel wafers) in patients with primary malignant glioma. Neuro Oncol. 2003;5(2):79–88. 12672279 10.1215/S1522-8517-02-00023-6 PMC1920672

[5] Fleming AB, Saltzman WM. Pharmacokinetics of the carmustine implant. Clinical pharmacokinetics. 2002;41(6):403–19. 12074689 10.2165/00003088-200241060-00002

[6] Bregy A, Shah AH, Diaz MV, Pierce HE, Ames PL, Diaz D, et al The role of Gliadel wafers in the treatment of high-grade gliomas. Expert Rev Anticancer Ther. 2013;13(12):1453–61. 10.1586/14737140.2013.840090 24236823

[7] Bobo RH, Laske DW, Akbasak A, Morrison PF, Dedrick RL, Oldfield EH. Convection-enhanced delivery of macromolecules in the brain. Proceedings of the National Academy of Sciences of the United States of America. 1994;91(6):2076–80. 8134351 10.1073/pnas.91.6.2076 PMC43312

[8] Mehta AI, Choi BD, Ajay D, Raghavan R, Brady M, Friedman AH, et al Convection enhanced delivery of macromolecules for brain tumors. Curr Drug Discov Technol. 2012;9(4):305–10. 22339074 10.2174/157016312803305951 PMC7521418

[9] White E, Bienemann A, Malone J, Megraw L, Bunnun C, Wyatt M, et al An evaluation of the relationships between catheter design and tissue mechanics in achieving high-flow convection-enhanced delivery. J Neurosci Methods. 2011;199(1):87–97. 10.1016/j.jneumeth.2011.04.027 21549753

[10] Saito R, Krauze MT, Noble CO, Tamas M, Drummond DC, Kirpotin DB, et al Tissue affinity of the infusate affects the distribution volume during convection-enhanced delivery into rodent brains: implications for local drug delivery. J Neurosci Methods. 2006;154(1–2):225–32. 16472868 10.1016/j.jneumeth.2005.12.027

[11] MacKay JA, Deen DF, Szoka FC Jr. Distribution in brain of liposomes after convection enhanced delivery; modulation by particle charge, particle diameter, and presence of steric coating. Brain Res. 2005;1035(2):139–53. 15722054 10.1016/j.brainres.2004.12.007

[12] Groothuis DR, Vavra MW, Schlageter KE, Kang EW, Itskovich AC, Hertzler S, et al Efflux of drugs and solutes from brain: the interactive roles of diffusional transcapillary transport, bulk flow and capillary transporters. Journal of cerebral blood flow and metabolism: official journal of the International Society of Cerebral Blood Flow and Metabolism. 2007;27(1):43–56. 10.1038/sj.jcbfm.9600315 16639426

[13] Barua NU, Lowis SP, Woolley M, O'Sullivan S, Harrison R, Gill SS. Robot-guided convection-enhanced delivery of carboplatin for advanced brainstem glioma. Acta Neurochir (Wien). 2013;155(8):1459–65. 23595829 10.1007/s00701-013-1700-6

[14] Barua NU, Hopkins K, Woolley M, O'Sullivan S, Harrison R, Edwards RJ, et al A novel implantable catheter system with transcutaneous port for intermittent convection-enhanced delivery of carboplatin for recurrent glioblastoma. Drug delivery. 2014. 10.3109/10717544.2014.908248 24786643

[15] Bienemann A, White E, Woolley M, Castrique E, Johnson DE, Wyatt M, et al The development of an implantable catheter system for chronic or intermittent convection-enhanced delivery. J Neurosci Methods. 2012;203(2):284–91. 10.1016/j.jneumeth.2011.10.002 22015599

[16] Barua NU, Woolley M, Bienemann AS, Johnson DE, Lewis O, Wyatt MJ, et al Intermittent convection-enhanced delivery to the brain through a novel transcutaneous bone-anchored port. J Neurosci Methods. 2013;214(2):223–32. 10.1016/j.jneumeth.2013.02.007 23419699

[17] Zhou J, Patel TR, Sirianni RW, Strohbehn G, Zheng MQ, Duong N, et al Highly penetrative, drug-loaded nanocarriers improve treatment of glioblastoma. Proc Natl Acad Sci U S A. 2013;110(29):11751–6. 10.1073/pnas.1304504110 23818631 PMC3718184

[18] Sawyer AJ, Saucier-Sawyer JK, Booth CJ, Liu J, Patel T, Piepmeier JM, et al Convection-enhanced delivery of camptothecin-loaded polymer nanoparticles for treatment of intracranial tumors. Drug Deliv Transl Res. 2011;1(1):34–42. 10.1007/s13346-010-0001-3 21691426 PMC3117592

[19] Yanamandra N, Kondraganti S, Srinivasula SM, Gujrati M, Olivero WC, Dinh DH, et al Activation of caspase-9 with irradiation inhibits invasion and angiogenesis in SNB19 human glioma cells. Oncogene. 2004;23(13):2339–46. 14767475 10.1038/sj.onc.1207406

[20] Birks SM, Danquah JO, King L, Vlasak R, Gorecki DC, Pilkington GJ. Targeting the GD3 acetylation pathway selectively induces apoptosis in glioblastoma. Neuro Oncol. 2011;13(9):950–60. 10.1093/neuonc/nor108 21807667 PMC3158019

[21] White E, Bienemann A, Pugh J, Castrique E, Wyatt M, Taylor H, et al An evaluation of the safety and feasibility of convection-enhanced delivery of carboplatin into the white matter as a potential treatment for high-grade glioma. Journal of neuro-oncology. 2012;108(1):77–88. 10.1007/s11060-012-0833-4 22476649

[22] White E, Bienemann A, Taylor H, Hopkins K, Cameron A, Gill S. A phase I trial of carboplatin administered by convection-enhanced delivery to patients with recurrent/progressive glioblastoma multiforme. Contemp Clin Trials. 2012;33(2):320–31. 10.1016/j.cct.2011.10.010 22101221

[23] Barua NU, Bienemann AS, Hesketh S, Wyatt MJ, Castrique E, Love S, et al Intrastriatal convection-enhanced delivery results in widespread perivascular distribution in a pre-clinical model. Fluids Barriers CNS. 2012;9(1):2 10.1186/2045-8118-9-2 22264361 PMC3274474

[24] Skinner EK, Price GJ. Encapsulation and release of aqueous components from sonochemically produced protein microspheres. Chem Commun (Camb). 2012;48(74):9260–2. 22875241 10.1039/c2cc34926d

[25] Parker KA, Pilkington GJ. Apoptosis of human malignant glioma-derived cell cultures treated with clomipramine hydrochloride, as detected by Annexin-V assay2006.

[26] Paxinos G, Watson C, Pennisi M, Topple A. Bregma, lambda and the interaural midpoint in stereotaxic surgery with rats of different sex, strain and weight. Journal of neuroscience methods. 1985;13(2):139–43. 3889509 10.1016/0165-0270(85)90026-3

[27] Stupp R, Hegi ME, Gilbert MR, Chakravarti A. Chemoradiotherapy in malignant glioma: standard of care and future directions. J Clin Oncol. 2007;25(26):4127–36. 17827463 10.1200/JCO.2007.11.8554

[28] Pardridge WM. Strategies for drug delivery through the blood-brain barrier. Neurobiol Aging. 1989;10(5):636–7; discussion 48–50. 2812242 10.1016/0197-4580(89)90160-7

[29] Bogdahn U, Hau P, Stockhammer G, Venkataramana NK, Mahapatra AK, Suri A, et al Targeted therapy for high-grade glioma with the TGF-beta2 inhibitor trabedersen: results of a randomized and controlled phase IIb study. Neuro-oncology. 2011;13(1):132–42. 10.1093/neuonc/noq142 20980335 PMC3018908

[30] Bruce JN, Fine RL, Canoll P, Yun J, Kennedy BC, Rosenfeld SS, et al Regression of Recurrent Malignant Gliomas with Convection-Enhanced Delivery of Topotecan. Neurosurgery. 2011. 10.1227/NEU.0b013e3182233e24 PMC4940854 21562434

[31] Barua NU, Lowis SP, Woolley M, O'Sullivan S, Harrison R, Gill SS. Robot-guided convection-enhanced delivery of carboplatin for advanced brainstem glioma. Acta Neurochir (Wien). 2013. 10.1007/s00701-013-1700-6 23595829

[32] Danhier F, Ansorena E, Silva JM, Coco R, Le Breton A, Preat V. PLGA-based nanoparticles: an overview of biomedical applications. J Control Release. 2012;161(2):505–22. 10.1016/j.jconrel.2012.01.043 22353619

[33] Govender T, Stolnik S, Garnett MC, Illum L, Davis SS. PLGA nanoparticles prepared by nanoprecipitation: drug loading and release studies of a water soluble drug. J Control Release. 1999;57(2):171–85. 9971898 10.1016/s0168-3659(98)00116-3

[34] Zhang Z, Lee SH, Gan CW, Feng SS. In vitro and in vivo investigation on PLA-TPGS nanoparticles for controlled and sustained small molecule chemotherapy. Pharmaceutical research. 2008;25(8):1925–35. 10.1007/s11095-008-9611-6 18509603

[35] Song XR, Zheng Y, He G, Yang L, Luo YF, He ZY, et al Development of PLGA nanoparticles simultaneously loaded with vincristine and verapamil for treatment of hepatocellular carcinoma. Journal of pharmaceutical sciences. 2010;99(12):4874–9. 10.1002/jps.22200 20821385

[36] Nance EA, Woodworth GF, Sailor KA, Shih TY, Xu Q, Swaminathan G, et al A dense poly(ethylene glycol) coating improves penetration of large polymeric nanoparticles within brain tissue. Sci Transl Med. 2012;4(149):149ra19. 10.1126/scitranslmed.3003594 PMC3718558 22932224

